# Idebenone: Novel Strategies to Improve Its Systemic and Local Efficacy

**DOI:** 10.3390/nano8020087

**Published:** 2018-02-05

**Authors:** Lucia Montenegro, Rita Turnaturi, Carmela Parenti, Lorella Pasquinucci

**Affiliations:** 1 Department of Drug Sciences, Pharmaceutical Technology Section, University of Catania, Viale A. Doria 6, 95125 Catania, Italy; 2 Department of Drug Sciences, Medicinal Chemistry Section, University of Catania, Viale A. Doria 6, 95125 Catania, Italy; rita.turnaturi@tiscali.it (R.T.); lpasquin@unict.it (L.P.); 3 Department of Drug Sciences, Pharmacology and Toxicology Section, University of Catania, Viale A. Doria 6, 95125 Catania, Italy; cparenti@unict.it

**Keywords:** idebenone, delivery systems, nanocarriers, idebenone analogues, neurodegenerative diseases

## Abstract

The key role of antioxidants in treating and preventing many systemic and topical diseases is well recognized. One of the most potent antioxidants available for pharmaceutical and cosmetic use is Idebenone (IDE), a synthetic analogue of Coenzyme Q_10_. Unfortunately, IDE’s unfavorable physicochemical properties such as poor water solubility and high lipophilicity impair its bioavailability after oral and topical administration and prevent its parenteral use. In recent decades, many strategies have been proposed to improve IDE effectiveness in the treatment of neurodegenerative diseases and skin disorders. After a brief description of IDE potential therapeutic applications and its pharmacokinetic and pharmacodynamic profile, this review will focus on the different approaches investigated to overcome IDE drawbacks, such as IDE incorporation into different types of delivery systems (liposomes, cyclodextrins, microemulsions, self-micro-emulsifying drug delivery systems, lipid-based nanoparticles, polymeric nanoparticles) and IDE chemical modification. The results of these studies will be illustrated with emphasis on the most innovative strategies and their future perspectives.

## 1. Introduction

Idebenone (IDE), synthesized in Japan in the 1980s, is an analogue of coenzyme Q_10_ (CoQ10), the lipophilic electron carrier and endogenous antioxidant found in all cellular mitochondrial membranes [1,2].

**Figure 1:**
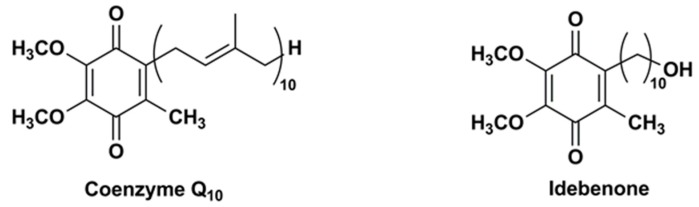
Chemical structures of CoQ10 and IDE.

Structurally, CoQ10 and IDE share the same substituted 1,4-benzoquinone ring but they have a different side chain at the carbon 2, i.e., IDE has a hydroxydecyl side chain (10 carbon atoms) while CoQ10 has a long side chain of 10 isoprene moieties (50 carbon atoms) (Figure 1). 

Despite the fact that the hydroxydecyl side chain makes IDE less lipophilic than CoQ10, its water solubility remains very low similar to the parent compound, thus preventing its administration in aqueous vehicles.

However, the hydroxydecyl side chain of IDE seems to be of the ideal length for favoring its partitioning into the mitochondrial membrane and for a better blood brain barrier (BBB) permeation in comparison with CoQ10 [3].

IDE acts as an electron carrier in the mitochondrial electron transport chain, thereby facilitating the production of ATP. At this site, although IDE inhibits complex I (NADH dehydrogenase), it acts as a potent antioxidant agent resulting effective at transferring electrons from complex II (succinate dehydrogenase) to complex III, but not from complex I to complex II [4].

Given its mechanism of action, IDE was employed for the treatment of neurodegenerative disorders and diseases that exhibited mitochondrial etiology [5]. IDE was tested with conflicting results in Friedreich’s ataxia (FA) [6], a genetic, progressive disease that usually begins in childhood and affects multiple systems, including the nervous system and the heart. FA is caused by deficits in mitochondrial production and expression of frataxin, a protein involved in iron metabolism and redox homeostasis [7,8]. 

In IDE-treated FA patients, generally, a reduction in oxidative stress markers was reported and many patients showed an improvement of non-neurological symptoms, as well. However, in a subsequent randomized, double-blind, placebo-controlled trial, no statistically significant difference between the placebo and IDE was revealed on the International Cooperative Ataxia Rating Scale [9,10]. 

Therefore, although initially “authorized with conditions” for the treatment of FA in Canada, under the trade name CATENA^®^, IDE was withdrawn from the market in 2013 for lack of efficacy [4].

Increasing evidence has indicated that mitochondrial dysfunctions display a significant role in the pathophysiological processes of the most prevalent neurodegenerative disorder, Alzheimer’s disease (AD) [11]. Preclinical studies reported that IDE produced neuroprotection against β amyloid induced neurotoxicity both in vitro and in vivo [12]. Clinical studies showed IDE neuroprotective effects in AD patients on AD Assessment Scale (ADAS) scores [13]. However, IDE failed to decrease AD associated cognitive decline in a larger multicenter, double-blind, placebo-controlled trial [14]. Thus, as in the case of FA, IDE approval to treat Alzheimer’s disease or related cognitive dementias has not been obtained.

In a recent animal study, IDE was found to protect hippocampal HT22 cells from in vitro glutamate-induced cell death, although IDE-treated experimental allergic encephalomielitis mice did not exhibit any significant inflammation, demyelination and axonal injury reduction [15]. Given that mitochondrial dysfunctions may play a key role in progressive axonal loss in Multiple Sclerosis (MS), further investigations on the possible therapeutic use of this drug for Primary Progressive MS (PPMS) are justified. A phase II trial of IDE efficacy in PPMS is currently underway (NCT01854359) [16]. 

Leber’s hereditary optic neuropathy (LHON,) a rare genetic mitochondrial disease that causes rapid and progressive bilateral vision loss, is the only mitochondrial disease for which IDE (Raxone^®^) has been approved by the European Medicine Agency to treat visual impairment in adolescents and adults [17,18]. IDE has shown to be especially promising for a subgroup of patients with the m.11778G > A gene mutation even if there is still uncertainty about the magnitude of the visual benefit when compared with the natural history of the disease [19].

Lekoubou et al. [20] suggested the long-term safety and potential benefit of oral administered l-arginine and IDE in the prevention of stroke-like episodes in adult patients with mitochondrial encephalopathy, lactic acidosis and stroke-like episodes (MELAS), a maternally inherited multisystem disorder. 

The remarkable clinical results from DELOS trial clearly showed that IDE could slow the loss of pulmonary function in patients with Duchenne muscular dystrophy. DELOS post-hoc analysis indicated that the protective effect of IDE on the respiratory function was associated with a reduced risk of bronchopulmonary complications and a reduced need for systemic antibiotic assumption [21]. 

A recent study shed light on the mechanism of action of IDE and provided more insights into its possible therapeutic use for treating or preventing atherosclerosis by ameliorating mitochondrial dysfunctions in endothelial cells [22]. 

Furthermore, IDE has been proposed for the treatment of skin disorders that could benefit from topical supplementation with antioxidants. Regarding its topical application, IDE showed the highest oxidative protection capacity when compared to dl-α-tocopherol, kinetin, CoQ10, l-ascorbic acid, and dl-α-lipoic acid [23]. Hence, the cosmetic industry has considered IDE an attractive molecule to develop anti-ageing products.

To date, all clinical trials on IDE efficacy in mitochondrial disorders and neuromuscular diseases have been carried out dosing IDE orally. After oral administration in healthy humans, IDE absorption by the gastrointestinal tract is fast but less than 1% of the administered dose reaches the systemic circulation, due to an extensive first-pass effect in the liver and intestinal mucosa [24]. Therefore, despite IDE ability to cross the BBB, its poor oral bioavailability, even after administration of high doses, could hinder this molecule from achieving therapeutic levels in the brain. 

To improve IDE physicochemical, pharmacokinetic and pharmaco-dynamic properties, several researchers investigated the feasibility of using suitable drug delivery systems and/or designed and synthesized IDE derivatives. In this review, the numerous strategies attempted to overcome IDE drawbacks, such as IDE incorporation into different types of delivery systems (liposomes, cyclodextrins, microemulsions, self-micro-emulsifying drug delivery systems, lipid-based nanoparticles, polymeric nanoparticles) and IDE chemical modifications will be illustrated, along with the results of in vitro and in vivo studies aimed at evaluating their potential applications to improve IDE efficacy. 

## 2. Drug Delivery Systems

In the last decades, many delivery systems have been designed and investigated to improve drug efficacy in comparison with conventional dosage forms. Since the first controlled release formulation introduced by Smith Kline and French in 1952 [25], three different generations of drug delivery systems have been developed [26]. The main goals of these innovative dosage forms were to enhance drug bioavailability and stability, to control efficiently drug release from the vehicle and to achieve a drug targeting effect, thus reducing unwanted side effects and improving patient compliance [27]. In the following sections, the most promising delivery systems assessed as carriers for IDE will be described, along with their features and their advantages and drawbacks. 

### 2.1. Liposomes

Liposomes are spherical vesicles resulting from the spontaneous aggregation of natural or synthetic phospholipids in aqueous media. As phospholipids form bilayer structures, these vesicles can incorporate both hydrophilic and lipophilic compounds: polar molecules can be dissolved in the aqueous core of the vesicles and lipophilic molecules can be located in the hydrophobic domains of the bilayer structures composed of phospholipid fatty acid chains [28]. Since their fortuitous discovery by Bangham et al. [29], liposome potential applications for pharmaceutical purposes have been recognized and their use as drug delivery systems has been widely reviewed [30,31,32,33]. 

Two different in vitro studies evaluated the protective effects of IDE loaded liposomes against ethanol-induced damages on astrocyte cell cultures [34,35]. In the first study, conventional liposomes were used as carriers for IDE [34] while in subsequent investigations IDE was incorporated in PEGylated large unilamellar vesicles (LUV) [35]. PEGylated liposomes were supposed to escape from binding to opsonins and not to be cleared by phagocytotic cells. Both studies showed improved cell viability and reduced ethanol-induced damages after treatment with IDE loaded liposomes. In these works, the authors suggested that IDE could be solubilized within the liposomal bilayer structure. Therefore, this approach could allow obtaining aqueous IDE formulations that could be used for parenteral administration, thus avoiding the first-pass effect that affects IDE oral dosing. The resulting improvement of IDE bioavailability could be expected to provide better outcomes in the treatment of neurological diseases.

Pignatello et al. [36] synthesized prodrugs of IDE with lipoamino acids and incorporated these IDE derivatives in neutral or negatively charged small unilamellar liposomes. Although the authors reported an increase of the protective effect against the oxidative stress induced on astrocyte cell cultures of IDE loaded liposomes in comparison with free IDE, these liposomal suspensions did not release IDE efficiently. The results of this study pointed out the need of choosing proper liposome features to obtain successful IDE delivery systems.

As lipid-based nanocarriers, including liposomes, could be used for topical delivery of antioxidants such as IDE [37], Shah et al. compared in vitro skin penetration and cytotoxicity of IDE from three different types of vesicles: conventional liposomes, cationic liposomes (Leciplex) and invasomes (flexible, neutral, phospholipid-based vesicles containing different types of phosphatidyl choline, terpenes, and ethanol) [38]. This work showed that Leciplex provided both the best skin penetration and the highest in vitro cytotoxicity on B16F10 melanoma cell lines, thus confirming the usefulness of properly designed liposomes as carriers for topical drug delivery. 

### 2.2. Cyclodextrins

Due to their structural features that make cyclodextrins (CDs) able to host different types of molecules forming inclusion complexes, these cyclic oligosaccharides, derived from the degradation of starch catalyzed by the cyclodextrin glycosyltransferase, have been widely investigated as drug carriers. CDs, consisting of six, seven or eight linked d-glucopyranose units (α, β and γ-CDs, respectively) form a rigid toroidal structure, which depends on the chair conformation of the glucopyranose units [39]. While CDs external surface is hydrophilic, their central cavity is hydrophobic and can host lipophilic molecules with different sizes. Therefore, encapsulating poor water-soluble drugs in CDs has been considered a useful tool to obtain an improvement of aqueous solubility, avoiding the use of organic solvents or other adjuvants, and to increase drug dissolution rate and bioavailability after oral administration [40,41]. As the inclusion of a “guest” molecule in the CDs cavity is a reversible process, a controlled release of the encapsulated active compound can be achieved. Other advantages of forming CDs inclusion complexes include drug protection from degrading process, decrease of tissue irritation (gastrointestinal and ocular mucosa, skin etc.) on drug direct contact, elimination of unpleasant odors or taste and prevention of drug-additive interactions [42,43,44,45]. Although early studies focused on naturally occurring CDs, at present numerous chemical modifications of the CDs are available and are preferentially used for pharmaceutical purposes due to their better technological and biological properties [46]. In particular, hydroxypropyl-β-cyclodextrins have been widely investigated as drug carriers because of their high water solubility and their ability to avoid the formation of crystalline cholesterol complexes that could lead to kidney damaging effects. An early study performed using β-cyclodextrins pointed out a notable increase of IDE aqueous solubility and dissolution rate after inclusion in these CDs in comparison with free IDE [47]. However, as β-cyclodextrins proved to be nephrotoxic, a further investigation was carried out using hydroxypropyl-β-cyclodextrins to form IDE inclusion complexes, showing results similar to those obtained using β-cyclodextrins [48]. 

Rathi et al. [49] performed in vitro experiments to improve IDE permeation through the bovine buccal mucosa using different penetration enhancers including IDE physical mixtures with β-cyclodextrins, hydroxypropyl-β-cyclodextrins, and a complex of IDE with hydroxypropyl-β-cyclodextrins. The authors observed the highest enhancement ratio (45.93) for the complex of IDE with hydroxypropyl-β-cyclodextrins, concluding that the inclusion complex could act as a permeation enhancer. Unfortunately, no data have been reported about the stability of this IDE inclusion complex. However, these results pointed to the feasibility of using complexes with hydroxypropyl-β-cyclodextrins to deliver IDE via the buccal or sublingual mucosa, thus avoiding the first-pass effect that strongly impair IDE oral bioavailability. 

Using 2-hydroxypropyl-β-cyclodextrins to form IDE inclusion complexes in the ratio 1:5–1:100 IDE:CDs, a Chinese patent [50] showed the possibility of preparing suitable parenteral formulations for clinical use. 

A recent study on IDE hydroxypropyl-β-cyclodextrins complexes was performed in an animal model of carrageenan-induced thermal hyperalgesia to assess the potential analgesic and anti-inflammatory activity of IDE [51]. As an oxidative stress is involved in inflammatory pain, antioxidants such as IDE could exert a beneficial effect. However, such activity was never clearly highlighted for IDE. The results of these experiments pointed out a significant anti-inflammatory and analgesic effect of this inclusion complex and led the authors to conclude that a suitable formulation could provide pharmacological effects that were not evident after treatment with the free drug. 

To improve IDE water solubility, Cannavà et al. [52] prepared IDE complexes with sulfobutyl ether-β-cyclodextrins. The authors fully characterized these complexes, reporting a remarkable increase of IDE water solubility due to the complexation with sulfobutyl ether-β-cyclodextrins and a subsequent significant enhancement of IDE dissolution rate. Therefore, the authors hypothesized that this approach could be expected to improve IDE bioavailability after oral administration, although no in vitro or in vivo data supported this conclusion. 

Recently, a soluble β-cyclodextrins polymer (β-CDs crosslinked with epichlorohydrin) in association with an enhancer of dissolution rate (carboxymethyl cellulose) was used to prepare spray-dried microparticles as carriers for IDE oral administration [53]. This strategy provided an improvement of IDE water solubility, wettability and dissolution rate compared to free IDE, suggesting that such microsystems could be regarded a useful tool to increase IDE absorption and bioavailability after oral dosing. 

### 2.3. Microemulsions and Self-Microemulsifying Drug Delivery System SMEDDS

The term “microemulsions” (MEs) has been used to define systems that share the same basic components with the emulsions (oils, surfactants and water) but differ because of their higher thermodynamic stability and their smaller droplet sizes (10–150 nm) [54]. MEs properties have prompt their use as carriers to improve water solubility of lipophilic drugs and to achieve controlled drug release for different administration routes [55,56,57,58,59]. MEs prepared using the phase inversion temperature (PIT) method and containing low percentages of surfactants were investigated as topical carriers for different active compounds including IDE [60]. This study revealed that IDE release from these MEs depended on the type of surfactant and on the lipophilicity of the oils used to prepare the colloidal system. Although these MEs were designed for topical application, they could be useful to develop IDE controlled release oral dosage forms, as well. 

To overcome one of the major drawbacks of MEs, i.e., their high water content, several researchers developed anhydrous MEs defined as self-microemulsifying drug delivery system (SMEDDS) [61,62]. These self-dispersing lipid formulations, consisting of drug, oils, surfactants and co-surfactants, are not themselves MEs, but they spontaneously form thermodynamically stable O/W MEs when mixed with the aqueous environments of the stomach. As these colloidal systems allow solubilizing poorly water-soluble drugs and avoid the dissolution step required for drug absorption from solid oral dosage forms, they have been mainly investigated to improve the oral bioavailability of lipophilic drugs. 

Kim et al. [63] prepared SMEDDS using different mixtures of oils and surfactants to increase IDE solubility and dissolution rate. The authors reported a two-fold increase of IDE dissolution rate from SMEDDS in comparison with conventional tablets, suggesting that these formulations could be expected to improve IDE bioavailability after oral administration. 

### 2.4. Polymeric Nanoparticles

Polymeric nanoparticles are regarded as one of the most promising strategies in the field of nanomedicine as they show several advantages compared to conventional formulations such as controlled drug release, increased drug solubility and absorption at the target site, biodegradability, good stability and tolerability [64,65]. Polymeric nanoparticles can be structurally different as they can consist of: (a) a uniform polymeric matrix dispersing or dissolving the active ingredient (nanospheres or matrix systems); (b) an oil core surrounded by a polymeric membrane with the drug dissolved in the liquid core or adsorbed at the nanoparticles surface, depending on its physicochemical properties (nanocapsules or reservoir systems) [66]. To prepare these nanoparticles, natural or artificial, biodegradable polymers are generally used and the most commonly are poly-l-lactic acid (PLA) and copolymers with glycolic acid (PLGA) [67].

IDE loaded nanocapsules were prepared using polyethyl-2-cyanoacrylates (PECA) and assessed for their in vitro antioxidant activity on human fibroblasts [68]. In this study, an increase of IDE water solubility after its loading into these PECA nanoparticles was observed along with a greater inhibition of radical oxygen species (ROS) production compared to free IDE. Although the authors suggested that these polymeric nanoparticles could be suitable to improve IDE oral delivery, the mechanisms involved in determining IDE loaded PECA nanocapsules antioxidant activity were not clearly elucidated as these IDE nanocarriers showed a different ability in inhibiting the oxidative stress induced by diethylmaleate (DEM) or by H_2_O_2_.

Chitosan nanoparticles have been designed to improve IDE topical delivery. Amorim et al. [69] assessed the stability, irritant effect and antioxidant activity of free IDE in comparison with IDE loaded into chitosan and *N*-carboxymethylchitosan nanoparticles. The authors observed an increased IDE stability and a decreased mucous membrane irritation loading IDE into these nanoparticles and IDE antioxidant activity was preserved after storage of these nanocarriers for 90 days. 

Glutathionylchitosan nanoparticles for topical co-administration of glutathione and IDE were investigated, showing high antioxidant activity without significant toxic effects on cultures of human keratinocytes [70]. Although both the above-mentioned studies suggested that IDE loaded chitosan nanoparticles could be suitable for IDE topical or nasal delivery, this conclusion was not supported by in vivo or in vitro data. 

### 2.5. Solid Lipid Nanoparticles and Nanostructured Lipid Carriers

In the 90s, solid lipid nanoparticles (SLNs) were developed to merge the advantages of liposomes and polymeric nanoparticles while overcoming their drawbacks. Ability to incorporate lipophilic and hydrophilic active compounds, long-term stability, improved bioavailability and stability of the entrapped molecules, controlled drug release, drug targeting, safety, low cost of production and easy scale-up are some of the features of these novel lipid-based nanocarriers [71,72].

SLNs are generally prepared from biocompatible solid lipids stabilized by different surfactants. The highly ordered packing of the lipid core of these nanocarriers could lead to low drug encapsulation efficiency and to drug leakage from the nanoparticles during preparation and storage because of lipid crystallization phenomena. To improve drug loading and to reduce drug expulsion from the carrier, a second generation of lipid nanoparticles, called nanostructured lipid carriers (NLCs), was developed by partially replacing solid lipids with liquid lipids [73,74,75]. As NLCs have a less organized lipid matrix, these nanocarriers could incorporate greater amounts of active compounds with minor problems of drug leakage. SLNs and NLCs are very versatile drug delivery systems as they can load various drugs and can be administered by different routes such as oral, parenteral and topical. In particular, these nanocarriers have been widely investigated for the delivery of antioxidants, including IDE [76,77]. Their features make SLNs and NLCs attractive carriers for drug delivery to the brain, as well [78]. Due to their small size, these nanoparticles could escape the reticuloendothelial system (RES) uptake, thus showing prolonged circulation into the blood. Several studies have evidenced greater efficacy of drugs after their loading into SLNs for the treatment of different brain diseases [78]. 

Two different studies were performed to evaluate the antioxidant activity of IDE loaded SLNs in primary cultures of astrocytes as model of brain tissue. Stancampiano et al. [79] prepared IDE loaded SLNs using the quasi-emulsion solvent diffusion (QESD) method. These nanocarriers showed good technological properties, prolonged IDE release and higher inhibition of 2, 2′-azobis-2-amidinopropane dihydrochloride (APPH)-induced lactate dehydrogenase (LDH) release in primary cultures of astrocytes compared to free IDE. However, their ability to reduce oxidative damages decreased as their concentration in the medium increased, likely because the lipids used to obtain these SLNs could induce ROS production. In a subsequent work [80], IDE loaded SLNs were prepared by the PIT method. These SLNs, made of nontoxic surfactants and a GRAS status lipid, showed good technological properties and were not cytotoxic. In addition, they were able to inhibit totally ROS production and to reduce markedly APPH induced LDH release in primary cultures of astrocytes. Permeation experiments on a model of BBB, consisting of MDCKIIMDR1 cell monolayers, showed that these SLNs allowed IDE permeation across a transcellular pathway [81]. Although the amount of IDE permeated, using these nanocarriers was about 0.50-fold lower than that obtained using free IDE, the authors highlighted that to perform in vitro experiments free IDE needed to be dissolved in a solvent (ethanol) unsuitable for parenteral administration while IDE loaded SLNs could be dispersed in a biocompatible aqueous medium. Therefore, the authors concluded that these SLNs could deserve further investigations as they could be regarded as an interesting approach to allow IDE parenteral administration. 

Due to their good technological properties, the same SLNs were investigated as carriers for IDE topical delivery [82]. The results of in vitro skin permeation studies pointed out an interesting targeting effect of IDE loaded SLNs because IDE accumulated into the upper skin layers (stratum corneum and epidermis) without permeating through the skin. In addition, the authors highlighted that the type of surfactant used to prepare these SLNs and IDE concentration in the colloidal dispersion affected this targeting effect. Li and Ge [83] compared in vitro IDE skin permeation from NLCs, nanoemulsions and oil vehicles, reporting a greater ability of NLCs to enhance IDE topical delivery and to improve its chemical stability. Various studies evaluated the interactions of IDE loaded lipid nanoparticles with biological membranes. A recent investigation on cellular interactions and photoprotective effects of IDE loaded NLCs showed a significant cellular uptake of IDE loaded NLCs in HaCaT cell lines and improved photoprotective effects [84]. Several differential scanning calorimetry studies were performed to evaluate the interactions with a model of biomembranes of both free IDE and IDE loaded SLNs [85,86,87,88]. The results of these experiments revealed IDE ability to interact with biomembranes and these interactions were affected by SLNs composition, thus confirming the need of choosing proper SLNs ingredients to favor IDE permeation across the biological barriers. 

To improve IDE ocular bioavailability, Leonardi et al. [89] prepared cationic SLNs to increase the pre-corneal residence time of the colloidal system by favoring SLNs interactions with the negatively charged ocular epithelium. The authors demonstrated that these IDE loaded SLNs showed good technological properties and suggested their potential use as carriers for IDE ocular delivery.

A very recent study [90] described a two-step approach aimed at improving IDE efficacy increasing its water solubility and its antioxidant activity. First, the authors synthesized IDE ester derivatives by covalent linking IDE to other two antioxidants, trolox (IDETRL) and lipoic acid (IDELIP) to obtain a synergic effect and then, they loaded these IDE derivatives into SLNs. Both IDE derivatives were more efficient antioxidants than IDE and their incorporation into SLNs prolonged their antioxidant activity. It is interesting to note that a remarkable increase of water solubility was obtained loading IDE and its derivatives into SLNs (400 folds for IDE, 780 folds for IDETRL, and 1600 folds for IDELIP). Owing to their very small sizes (23–25 nm), these SLNs could be supposed to escape easily the RES uptake and to be able to permeate across the BBB. Therefore, these results suggested that this approach could allow obtaining IDE aqueous formulations suitable for parenteral administration, which could improve IDE outcome in the management of neurodegenerative diseases.

All IDE delivery systems investigated to date have been summarized in Table 1.

## 3. Idebenone Analogues

As a strategy to improve physicochemical properties of IDE, modifications of the redox core of IDE have been evaluated. 

**Figure 2:**
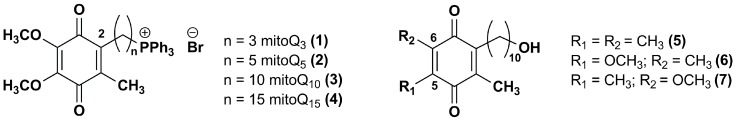
Chemical structures of IDE analogues modified at carbon 2 or 5 and/or 6.

Duveau et al. [91] synthesized two series of IDE analogues (Figure 2) to delineate the structural determinants responsible for the oxygen consumption in the mitochondrial respiratory chain. The first series of IDE analogues included triphenylphosphonium derivatives with an increasing side chain length (**1**–**4**), whereas the other series was characterized by the replacement of one or both methoxyl groups in position 5 and 6 of the 1,4-benzoquinone ring with methyl groups (**5**–**7**).

The increase of side chain length in triphenylphosphonium derivatives (**1**–**4**) inhibited the oxygen consumption in in vitro assay. On the contrary, IDE analogues **5**–**7** displayed a significant and comparable capability to stimulate the mitochondrial oxygen consumption, although lower than that of IDE, probably by supporting maximal electron transport activity. In CEM leukemia cells treated with DEM, which depleted cellular glutathione and produced ROS, compound **5**, with two methyl groups in lieu of IDE methoxyl groups, resulted the most effective and dose-dependent cytoprotective agent (77.3% viable cells at 0.5 μM).

**Figure 3:**
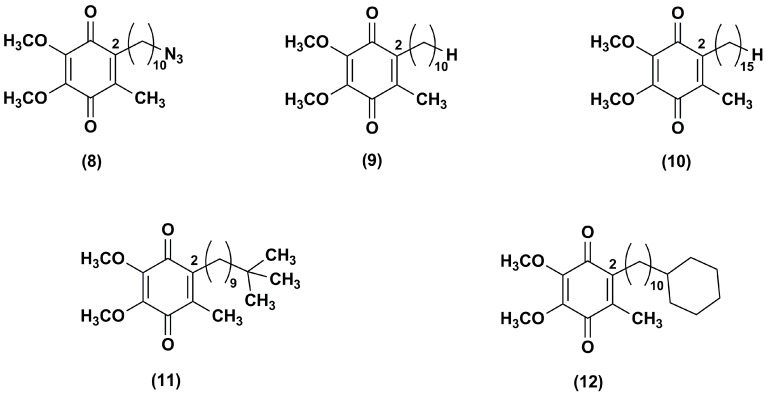
Chemical structures of IDE analogues with side chain modifications at position 2.

Fash et al. [92] explored the influence of the alkyl side chain nature through the synthesis of IDE derivatives bearing, at the position 2 of 1,4-benzoquinone ring, a 10-azidodecyl (**8**), decyl (**9**), pentadecyl (**10**), 9,9-dimethyldecyl (**11**) or 10-cyclohexyldecyl (**12**) chain in lieu of the 10-hydroxydecyl chain (Figure 3).

Differently from IDE, which contained a terminal polar alkyl side chain and inhibited the NADH oxidase and NADH ubiquinone oxido-reductase activity, compounds **8**–**10**, containing apolar chains, did not show the same activity. Derivatives **11**–**12**, containing a branched alkyl and a bulk side chain, respectively, showed a slight higher inhibitory effect versus NADH oxidase and NADH ubiquinone oxido-reductase in comparison to compounds **8**–**10**, although lower than that of IDE. In addition, all tested compounds increased the mitochondrial oxygen consumption in RGC-5 cells. Compounds **8**–**12** resulted more effective ROS scavengers (at 1 μM 71%, 80%, 100%, 88% and 100%, respectively) than IDE (at 1 μM 45%) in CEM leukemia lymphocyte cells treated with DEM. Furthermore, the same in vitro model pointed out the cytoprotective effects of compounds **9**, **10** and **12** (at 0.1 μM, 69%, 82% and 76% viable cells, respectively). These effects were more pronounced in comparison with IDE (at 0.1 μM, 25% viable cells) while compounds **8** and **11** were less active (at 0.1 μM, 11% and 9% viable cells, respectively). Among IDE derivatives, the increased hydrophobicity of the side chain in compounds **10** and **12** provided an improved activity that could reflect an increase of bioavailability.

**Figure 4:**
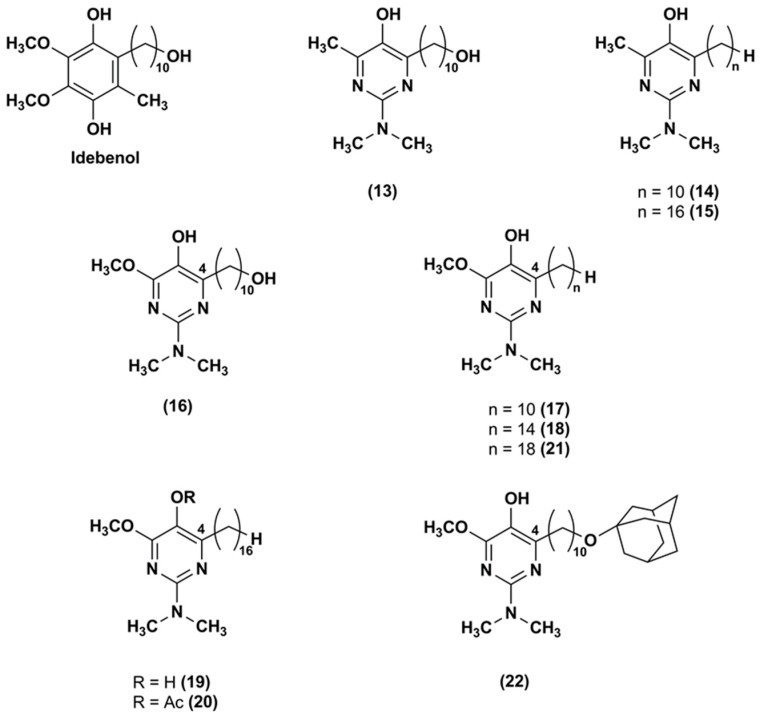
Chemical structures of the IDE aza-analogues.

To attenuate the effects of ROS produced by the respiratory chain, an aza-analogue (**13**) of IDE (Figure 4), with a pyrimidinol core in lieu of the 1,4-benzoquinone core, was synthesized and evaluated [93].

The aza-derivative (**13**) was more effective than α-tocopherol, IDE and idebenol at decreasing lipid peroxidation. This evidence was corroborated by the aza-derivative capability to protect mouse mitochondria membrane by inhibiting also the thiobarbituric acid-reactive substances production. In comparison to the parental ligands, the aza-compound (**13**) suppressed the ROS production at 5 μM in CEM leukemia cells treated with DEM. Its cytoprotective effects (EC_50_ = 250 nM) were three-fold higher than that of IDE and idebenol (EC_50_ = 820 and 720 nM, respectively). Similarly, compound **13** (EC_50_ = 390 nM) was more effective than IDE and idebenol (EC_50_ = 710 and 1030 nM, respectively) at protecting FA fibroblasts from oxidative stress induced by the glutathione synthesis inhibitor L-buthionine (*S*,*R*)-sulfoximine.

Through a synthetic pathway reported elsewhere [94,95], modifications of the side chain at the pyrimidinol core led to other two IDE derivatives (**14**, **15**) [96], both lacking of the terminal hydroxyl group at the side chain (Figure 4). The compound **14** bears the alkyl side chain of the experimentally largely used decylubiquinone, while the compound **15** has a longer chain (16 atoms). In comparison to IDE, both derivatives were more efficient in suppressing ROS. Despite the hydroxyl removal from alkyl side chain, the capability of compound **14** to inhibit the mitochondrial complex I (IC_50_ = 2 μM) and NADH oxidase was retained. However, compound **15**, with a longer side chain, did not show the same capability, suggesting that lipophilic side chain optimization was crucial for a possible therapeutic use. Both compounds were tested in several cell lines from patients with mitochondrial neurodegenerative diseases, such as LHON, Alzheimer’s and Parkinson’s disease lymphocytes, and behaved as better cytoprotective agents than IDE.

To improve the antioxidant activity, pyrimidinol derivatives (**16**–**22**), with a methoxyl group in orto to the phenolic hydroxyl group and with various side chains of different length in position 4, were synthesized (Figure 4) [97]. In glutathione depleted FA lymphocytes, all tested compounds resulted effective at reducing lipid peroxidation. In particular, the increased capability of the compound **16** to quench lipid peroxidation seemed to be due to the presence of the electron-donor methoxyl group. An influence of the side chain length was also reported. The derivatives with 14–16 atoms side chain (**18**–**19**) showed higher antioxidant activity in comparison with the derivative with longer side chain (18 atoms, **21**). In FA fibroblasts, an analogous antioxidant trend was reported with EC_50_ values (ranging from 22 to 345 nM) improved compared to IDE (EC_50_ = 551 nM) and aza-derivative (**13**) (EC_50_ = 456 nM). Thus, optimization of the redox core and the hydrophobic side chain may afford compounds with improved protective properties.

**Figure 5:**
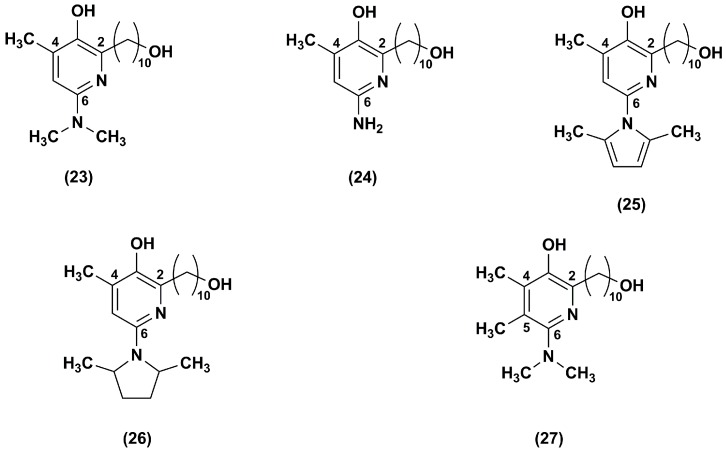
Chemical structures of IDE analogues with pyridinol core.

The same research group [98] synthesized a series of IDE derivatives with (a) a pyridinol core variously substituted in lieu of the IDE 1,4-benzoquinone; (b) the same IDE side chain at position 2 of the ring; (c) an amino group in position 6; and (d) a methyl group in position 4 or in 4 and 5 (**23**–**27**) (Figure 5).

As detected in FA lymphocytes, depleted of glutathione by treatment with DEM, all analogues (**23**–**27**), with exception of compound **25**, inhibited lipid peroxidation. In particular, among the series, analogues **26** and **27** were the most effective at quenching the lipid peroxidation, whereas compound **25** was the least efficacious. Moreover, in glutathione depleted CEM leukemia cells, all tested compounds (**23**–**27**) were more effective to suppress ROS than IDE, with the exception of compounds **24** and **25**. Compound **26** was the most potent ROS scavenger followed by compound **27**. Their cytoprotective effects were 2.5 and 1.2 times higher than that of IDE in DEM-treated CEM leukemia cells. Similarly, in FA lymphocytes treated with DEM, compounds **26** (EC_50_ = 0.15 μM) and **27** (EC_50_ = 0.56 μM) were more efficient in comparison to IDE (EC_50_ = 0.73 μM). Moreover, all synthesized derivatives, with the exception of compound **27**, showed a lower attitude to inhibit the NADH oxidase activity. These data revealed the importance of the amino group at position 6. Indeed, to elicit antioxidant and cytoprotective effects, a primary or tertiary aliphatic amino group was preferable rather than a tertiary aromatic amino group. The highest ability of compound **26** to stabilize the radicals formed during lipid peroxidation quenching was probably due to its electron-donating substituent at position 6 of the pyridinol core. 

**Figure 6:**
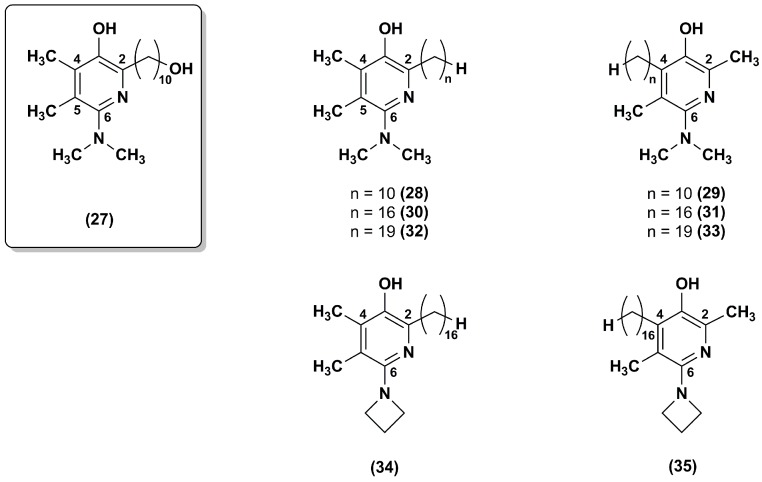
Chemical structures of compound **27** analogues.

A series of regioisomeric pairs of pyridinol derivatives of compound **27**, with different side chain lengths and lacking of the hydroxyl group, was synthesized (**28**–**33**). In addition, the authors obtained compounds **34** and **35** by introducing a 16 carbon atoms side chain and an azetidine group at position 6 of the pyridinol redox core (Figure 6) [99]. 

All synthesized derivatives showed less inhibitory effects on NADH oxidase activity than compound **27**. In compounds **30**–**35,** the presence of side chains with 16 or 19 carbon atoms withdraw almost completely their inhibitory effect. According to the data obtained for pyrimidinol derivatives [97], compounds **30**, **31**, **34** and **35**, with a 16 carbon atoms side chain at position 2, had better capacity to inhibit lipid peroxidation. Moreover, in comparison to the lead compound **27**, both regioisomeric compounds **30** and **31** were more effective at reducing ROS and at preventing mitochondrial depolarization under oxidative stress. Compounds **34** and **35**, with a 16 carbon atoms side chain and an azetidine group, were slightly less effective. Therefore, these data confirmed that the hydrophobic nature of the side chain had beneficial effects in different assays predictive of cytoprotective behavior in cells. Compounds **34** and **35,** with an azetidine group in position 6, displayed an improved metabolic stability with ~95% recovery after a 30 min microsomal incubation as assessed by in vitro microsomal studies.

**Figure 7:**
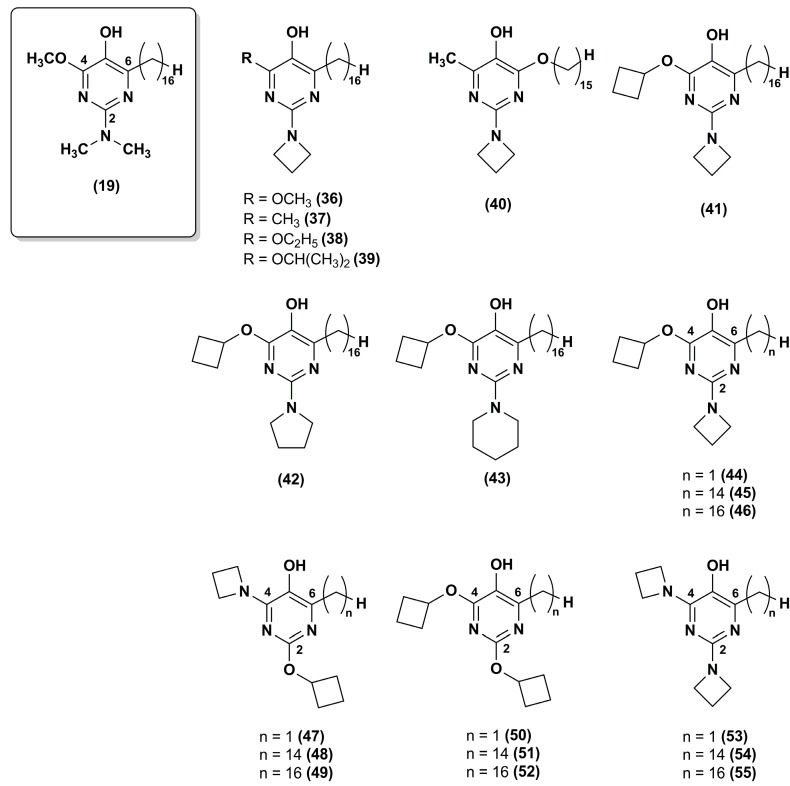
Chemical structures of compound **19** analogues.

To improve the stability of the lead compound **19** against the oxidative metabolism, Chevalier et al. [100] synthesized a series of derivatives modified at positions 2 and 4 of the pyrimidinol core (**36**–**43**) that were targets of oxidative dealkylation (Figure 7).

The compounds with exocyclic azetidine at position 2 (**36**–**41**) showed improved metabolic stability as reported for compounds **34** and **35** in the pyridinol series [99]. In addition, more sterically hindered ethers, such as that containing an isopropoxy moiety (**39**) at position 4, resulted in additional improvement of metabolic stability. Cyclic amines larger than azetidine (compounds **42** and **43**) compromised the antioxidant properties. Thus, the best analogue in this series was compound **39** that had good metabolic stability (76% recovery respect to 46% for compound **19**) and excellent cytoprotective properties at 0.5 μM.

Recently, the same research group synthesized twelve new analogues through a synthetic approach used elsewhere [101,102]. IDE analogues included 4-alkoxy-2-alkylamino derivatives (**44**–**46**) and their 2-alkoxy-4-alkylamino regioisomers (**47**–**49**), the 2,6-dialkoxy (**50**–**52**) and 2,6-dialkylamino analogues (**53**–**55**) (Figure 7) [103]. Compounds **45**–**46** and **48**–**49** showed high efficacy in suppressing lipid peroxidation and ROS, while dialkoxy (**50**–**52**) or dialkylamino (**53**–**55**) compounds did not show such activity. The presence of a hydrophobic side chain in position 6 was essential as no lipid peroxidation quenching activity was observed for compounds **44** and **47**. Moreover, compounds **48**–**49** had high metabolic stability with a good recovery (77% and 72%, respectively). Overall, the regioisomers in which the alkylamine substituent was at position 4 (**48**–**49**) were more effective than the regioisomers in which the alkylamine was at position 2 (**45**–**46**), providing a further improvement for pyrimidinol-based compounds. Thus, compound **48** was superior to any previously investigated pyrimidinol analogue.

**Figure 8:**
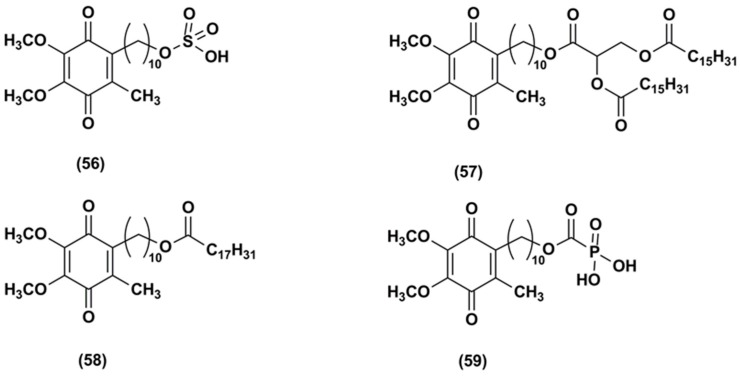
Chemical structures of carboxylic acid-substituted IDE derivatives.

In 2004, the Patent US6756045 B1 [104] reported the synthesis of a hydrophilic IDE ester, IDE sulfonic acid (**56**, Figure 8) and a method for preventing and treating cutaneous alterations applying a topical preparation. This formulation contained IDE, or IDE sulfonic acid, or a combination of IDE and IDE sulfonic acid. According to this patent, the use of IDE together with IDE sulfonic acid as anti-oxidant, free-radical absorber, stabilizer of mitochondrial membranes, stimulator of vesicular breathing and anti-apoptotic agent in cosmetic or dermatological preparations could improve IDE topical effectiveness.

The Patent US8173703 B2 reported the synthesis and in vivo test of carboxylic acid-substituted IDE derivatives (Figure 8) as antioxidant agents for skin treatment [105].

For instance, IDE dipalmitoyl glycerate (**57**) formulated in a cream was tested in 27 healthy human volunteers. This compound was able to reduce skin irritation and inflammation and the presence of carboxylic functions increased its skin permeability. Other derivatives such as IDE linoleate (**58**) and IDE phosphate (**59**) esters did not produce the same benefits, causing skin sensitization.

## 4. Conclusions

Due to IDE poor bioavailability after oral and topical administration, many researchers attempted different strategies to increase IDE efficacy in the treatment of neurodegenerative diseases and skin disorders. IDE chemical modifications mainly focused on increasing IDE activity at cellular or mitochondrial level while nanotechnology approaches aimed at designing novel formulations to improve IDE systemic and local efficacy. The encouraging results obtained loading IDE in different delivery systems such as liposomes, cyclodextrins and lipid-based nanoparticles could open new perspectives in the therapeutic outcomes of this strong antioxidant agent. 

## Figures and Tables

**Table 1:** Idebenone (IDE) delivery systems investigated to date. Ref. = reference.

Delivery System	Target	Investigated Properties and Results	Ref.
Liposomes	Brain delivery	IDE loaded liposomes reduced ethanol-induced injury on rat astroglial cell cultures and improved cell viability compared to free IDE. These liposomes were not suitable for in vivo systemic administration due to their uptake by the reticulo-endothelial system.	[34]
Liposomes (PEGylated large unilamellar vesicles)	Brain delivery Systemic administration	IDE loaded liposomes were more effective than the free drug in reducing ethanol-induced injury in rat primary cortical astrocyte cultures. A concentration-dependent toxic effect on cortical astrocytes was observed. These liposomes were supposed to be suitable for in in vivo systemic administration as they could escape the uptake by the reticulo-endothelial system.	[35]
Liposomes (neutral or negatively charged small unilamellar vesicles) loaded with IDE or IDE prodrugs	Brain delivery	These liposomes showed poor release of the encapsulated IDE prodrugs. Negative liposomes loading IDE and IDE prodrugs were less effective at reducing lactic dehydrogenase production and at protecting against oxidative damages in rat astrocyte cultures than the corresponding neutral liposomes.	[36]
Liposomes (conventional, cationic, invasome)	Skin delivery Topical administration	Cationic liposomes provided the highest IDE skin delivery in ex vivo human skin penetration studies and the highest in vitro cytotoxicity on B16F10 melanoma.	[38]
β-cyclodextrins	Not specified	IDE inclusion in β-cyclodextrins showed a linear increase in drug solubility and an enhancement of dissolution rate in comparison with free IDE.	[47]
Modified-β-cyclodextrin Dimethyl-β-cyclodextrin Hydroxypropyl-β-cyclodextrins	Brain delivery Oral administration	Dimethyl-β-cyclodextrins and hydroxypropyl-β-cyclodextrins showed the best ability to increase IDE water solubility and to enhance IDE dissolution rate.	[48]
β-cyclodextrins hydroxypropyl-β-cyclodextrins	Brain delivery Buccal administration	Cyclodextrins enhanced IDE water solubility, dissolution rate and permeability through the buccal mucosa. IDE complex with hydroxypropyl-β-cyclodextrins acted as a penetration enhancer for IDE buccal delivery, providing the best enhancement ratio (45.93) in comparison with all other penetration enhancers tested.	[49]
Hydroxypropyl-β-cyclodextrins	Systemic administration	Enhanced IDE solubility in aqueous vehicles	[50]
Hydroxypropyl-β-cyclodextrins	Systemic administration	Intraperitoneal pretreatment with hydroxypropyl-β-cyclodextrins complexed IDE inhibited hyperalgesia and edema in an animal model (rat) of carrageenan induced thermal hyperalgesia. This complex highlighted the analgesic and anti-inflammatory activity of IDE.	[51]
Sulfobutyl ether-β-cyclodextrins	Brain delivery Oral administration	IDE complexation with sulfobutyl ether-β-cyclodextrins increased its water solubility and dissolution rate.	[52]
β-cyclodextrins polymer	Brain delivery Oral administration	Loading IDE into microparticles containing a β-cyclodextrins polymer and an enhancer of dissolution rate increased its water solubility, wettability and dissolution rate.	[53]
Microemulsions	Skin delivery Topical administration	IDE release depended on the type of surfactant and on the lipophilicity of the oils used to prepare the microemulsion.	[60]
Self-microemulsifying drug delivery systems	Brain delivery Oral administration	IDE release rate from optimized SMEDDS was two-fold higher than that obtained from conventional tablets.	[63]
Polymeric nanoparticles (polyethyl-2-cyanoacrylates)	Brain delivery Oral and systemic administration	IDE loaded nanoparticles showed higher in vitro antioxidant effects in human fibroblasts than free IDE.	[68]
Polymeric nanoparticles (chitosan and *N*-carboxymethylchitosan)	Skin or nasal delivery Topical administration	These nanoparticles increased IDE stability while preserving its in vitro antioxidant activity and reducing mucous membrane irritation in comparison with the free drug.	[69]
Polymeric nanoparticles (Glutathionylchitosan)	Skin delivery Topical administration	IDE loaded nanoparticles showed a strong in vitro antioxidant activity while IDE in aqueous vehicle showed no activity. These nanoparticles were not cytotoxic in human keratinocytes (HaCaT) cell lines.	[70]
Solid lipid nanoparticles	Brain delivery Systemic administration	These solid lipid nanoparticles provided a slow and prolonged IDE in vitro release and maintained or increased IDE protective effect against free radical-induced oxidative stress in astrocyte cell cultures.	[79]
Solid lipid nanoparticles	Brain delivery Systemic administration	IDE in vitro release from these carriers depended on the type of surfactant used and the amount of loaded drug. IDE loaded solid lipid nanoparticles were more effective than free IDE at inhibiting free radical-induced oxidative stress in primary cultures of astrocytes obtained from rat cerebral cortex.	[80]
Solid lipid nanoparticles	Brain delivery Systemic administration	IDE permeability across a model of blood brain barrier (MDCKII-MDR1 cell monolayers) from these solid lipid nanoparticles was slightly lower than free IDE but IDE could be administered in aqueous media.	[81]
Solid lipid nanoparticles	Skin delivery Topical administration	These solid lipid nanoparticles provided an accumulation of IDE into the upper skin layers without any significant permeation through pig skin, depending on their composition and IDE loading.	[82]
Solid lipid nanoparticles (cationic)	Ocular delivery Topical administration	Cationic solid lipid nanoparticles provided an increase of IDE stability in comparison with the free drug while preserving its in vitro antioxidant activity.	[89]
Solid lipid nanoparticles IDE and IDE derivatives	Brain delivery Systemic administration	IDE and IDE derivatives loaded solid lipid nanoparticles showed prolonged in vitro antioxidant activity and increased water solubility.	[90]
Nanostructured lipid carriers	Skin delivery Topical administration	IDE loaded nanostructured lipid carriers increased IDE in vitro permeation through guinea pig skin and improved IDE chemical stability.	[83]
Nanostructured lipid carriers	Skin delivery Topical administration	IDE loaded nanostructured lipid carriers increased in vitro IDE skin deposition and cellular uptake (HaCaT cells), showing photo-protective effects against UVB-mediated oxidative stress in HaCaT cells.	[84]
